# Editorial: Challenges and advances in revision total joint arthroplasty

**DOI:** 10.1186/s42836-025-00298-y

**Published:** 2025-03-04

**Authors:** Sumon Nandi, Eryou Feng

**Affiliations:** 1 Department of Orthopaedics, University of Maryland School of Medicine, Baltimore, MD 21201 USA; 2 Department of Orthopaedics, Fujian Medical University Union Hospital, Fuzhou, 350001 China

**Keywords:** Hip, Knee, Joint, Arthroplasty, Revision

## Abstract

Revision total joint arthroplasty (TJA) is widely performed, and its incidence is increasing exponentially over time. Morbidity, mortality, as well as cost, both to the patient and the healthcare system, are significantly greater with revision TJA than primary TJA. Thus, efforts to minimize all-cause revision surgery are essential. In this special issue, we present articles on revision TJA epidemiology, surgical techniques, novel technology, implant design, and outcome optimization.

## Introduction

Revision total joint arthroplasty (TJA) is widely performed, and its incidence is increasing exponentially over time [1]. Though return to the operating room is undesirable for both surgeon and patient, revision for aseptic loosening is anticipated as the limit of TJA survival is approached. However, revision for periprosthetic joint infection (PJI), component malposition, periprosthetic fracture, instability, knee stiffness, or taper corrosion are unanticipated and represent fertile opportunities for research advances. Morbidity, mortality, as well as cost, both to the patient and the healthcare system, are significantly greater with revision TJA than primary TJA [2–5]. Thus, efforts to minimize all-cause revision surgery are essential.

Herein, we offer manuscripts that seek to minimize the incidence, and optimize the outcomes, of revision TJA.

## Summary of the Included Studies

Kabu et al. conducted a computed tomography (CT)-based study of 50 knees comparing bone coverage between varus-valgus constrained revision total knee arthroplasty (TKA) tibial component designs [6]. The authors concluded that asymmetric revision tibial components provided greater bone coverage than symmetric trays.

In a single-institution study of 10,202 cemented primary TKAs across multiple implant manufacturers, there was no increased risk of aseptic loosening or radiolucencies associated with Depuy Attune components [7]. However, it should be noted that mean follow-up for the Attune was approximately half as long as that of all other implants.

A case series of 27 femoral and tibial titanium metaphyseal cones implanted with a free-hand burring technique demonstrated no cases of aseptic loosening at mean 51-month follow-up [8]. If bone size or canal anatomy prevent metaphyseal cone preparation with cannulated reamers, this alternate technique is feasible.

Mohammad et al. performed a literature review of 5 articles on revision total hip arthroplasty (THA) patient expectations, which were exceedingly high and unrealistic for postoperative pain and function [9]. Though the quality of reviewed studies was limited, the importance of preoperative management of revision THA patient expectations is underscored.

A single-institution retrospective cohort study of 426 revision TJA patients concluded that trauma, with or without fracture, prior to revision surgery results in significantly higher risk of postoperative PJI or aseptic re-revision [10]. As a result, measures to minimize the risk of PJI, dislocation, and other mechanical adverse events is critical in revision TJA patients with preoperative history of trauma.

In a United States (U.S.) database study of 17,868 revision THA patients, higher modified frailty index (MFI) scores were associated with increased risk of adverse events and hospital readmission [11]. Preoperative utilization of the authors’ 8-item MFI may help predict, and potentially mitigate, postoperative risk following revision THA.

Shaarani et al., in a single-institution case series of 59 revision THAs for Paprosky I through IIIB defects, reported no cup revisions at mean 25-month follow-up when a porous titanium acetabular component with variable angle locking screws was utilized [12]. Use of this novel design acetabular shell yielded promising results at short-term follow-up.

A U.S. database query of 465,968 revision TKAs from 2006 to 2015 showed a 28.8% increase in incidence over time, most commonly for PJI, and 28.8% rate of postoperative adverse events [13]. This same group, in a U.S. database study of 400,974 revision THAs from 2006 to 2015, found a 28.5% increase in incidence over time, most commonly for instability, and a 39.5% rate of postoperative adverse events [14]. These findings highlight the importance of meticulous intraoperative technique in primary TJA and further optimization of preoperative, intraoperative, as well as postoperative revision TJA protocols.

## Conclusion

In this special issue, we present articles on revision TJA epidemiology, surgical techniques, novel technology, implant design, and outcome optimization. We, together with the manuscript authors, have aimed to provide guidance to the *Arthroplasty* readership in navigating the challenging clinical scenarios associated with revision TJA.

## Data Availability

Not applicable.
